# Autophagy in hepatic macrophages can be regulator and potential therapeutic target of liver diseases: A review

**DOI:** 10.1097/MD.0000000000033698

**Published:** 2023-05-12

**Authors:** Jun Ge, Hao Li, Jia-Qi Yang, Yuan Yue, Sheng-Yu Lu, Hong-Yun Nie, Tao Zhang, Pei-Ming Sun, Hong-Feng Yan, Hong-Wei Sun, Jian-Wu Yang, Jin-Lian Zhou, Yan Cui

**Affiliations:** a Department of General Surgery, Strategic Support Force Medical Center, Beijing 100101, China; b Department of General Surgery, The 306th Hospital of PLA-Peking University Teaching Hospital, Beijing 100101, China; c Department of Pathology, Strategic Support Force Medical Center, Beijing 100101, China.

**Keywords:** autophagy, kupffer, hepatic macrophages, liver diseases, polarization, inflammasomes

## Abstract

Hepatic macrophages are a complex population of cells that play an important role in the normal functioning of the liver and in liver diseases. Autophagy, as a maintainer of cellular homeostasis, is closely connected to many liver diseases. And its roles are not always beneficial, but manifesting as a double-edged sword. The polarization of macrophages and the activation of inflammasomes are mediated by intracellular and extracellular signals, respectively, and are important ways for macrophages to take part in a variety of liver diseases. More attention should be paid to autophagy of hepatic macrophages in liver diseases. In this review, we focus on the regulatory role of hepatic macrophages’ autophagy in a variety of liver diseases; especially on the upstream regulator of polarization and inflammasomes activation of the hepatic macrophages. We believe that the autophagy of hepatic macrophages can become a potential therapeutic target for management of liver diseases.

## 1. Introduction

As we all know, hepatic macrophages are a class of macrophages that lives in liver, with various origins and huge quantity, playing an important role in both disease and normal state. Hepatic macrophages has been found to participate in various liver disease such as liver injury, ischemia reperfusion injury, non-alcoholic steatohepatitis (NASH), alcoholic liver disease (ALD), viral hepatitis, etc.^[1]^ Hence, it is worthwhile to concentrate on hepatic macrophages to find potential therapeutic target.

Autophagy is a process that cells achieve self-protection and homeostasis. Studies shows that the autophagy plays a protective role on cells. Meanwhile, it may bring adverse consequences. Upon regulation and functional roles, the autophagy manipulates polarization and activation of inflammasomes to affect the progress of liver diseases. Polarization means the activation of macrophages stimulated by external environment, which will be involved in diseases development. Similarly, the activation of inflammasomes refers to the formation of multiprotein complex in the cells, which will further promote the release of cytokines and pyroptosis. By elaborating complex regulatory functions of hepatic macrophages’ autophagy in many liver diseases, we believe it can be a promising target for liver diseases treatment. Notably, the regulation of polarization and inflammasomes of autophagy are 2 critical functions, by which hepatic autophagy regulates the progression of liver diseases.

## 2. Hepatic macrophages

Macrophages, as a kind of myeloid immune cell widely distributed in all parts of the body, play a significant role in regulating inflammatory processes. Their primary function is to recognize, engulf and degrade harmful substance such as cellular debris, foreign material, or pathogens. Hepatic macrophages are macrophages existing in liver, which can maintain the hemostasis in both normal liver and liver disease. According to its origin, hepatic macrophages can be divided in to Kupffer cells (KCs, liver resident macrophages) and monocyte-derived macrophages (recruited macrophages).^[2]^ KCs, named after Karl Wilhelm von Kupffer, a German anatomist, originates from yolk sac and possesses self-renewal capacity independent of hematopoietic stem cells. Meanwhile, circulatory monocytes give rise to monocyte-derived macrophages, whose precursor cells is bone marrow–resident hematopoietic stem cells.^[3]^ What’s more, Wang et al^[4]^ found that when liver injury happens, peritoneal macrophages can migrate into injured place to contribute to tissue repair. However, other study also shows that when KCs is exhausted, monocytes will take the place of KCs, which displays the same phenotype with KCs.^[5]^

Furthermore, hepatic macrophages have different cell surface markers. In mice, KCs’ markers are CD11blow, F4/80hi, Clec4F+, CD68+, CX3CR1-, while monocyte-derived macrophages are CD11blow, F4/80hi, Ly6C+, CSF1R+. In human, KCs’ marker is CD68+, and monocyte-derived macrophages’ makers are CD14+, CCR2+.^[1]^ Circulating monocyte can also be divided into 2 subtypes: Ly-6Chi, Ly-6Clow. The character of Ly-6Chi is infiltrating tissue, while the hallmark feature of the later is patrol,^[6]^ so hepatic macrophages are not a simple type of cells but a kind of macrophages with heterogeneity.

Hepatic macrophages play an important role in both normal conditions and disease. The liver is the body’s reservoir of macrophages in mammalian body, because KSC comprises 80% to 90% total tissue macrophages, not to mention hepatic macrophages from circulating monocytes.^[7]^ It is found that about 20 to 40 macrophages partner every 100 hepatocytes in the healthy liver of rodent.^[8]^ KCs also account for approximately 25% hepatic nonparenchymal cells (including KCs, lymphocyte, stellate cell and endothelial cell as well as biliary cell).^[9]^ As a member of reticuloendothelial system in the liver, KCs mainly locates on the luminal side of the sinusoidal vessels. For this reason, KCs forms the first line of defense, which is exposed to substances from the intestine such as bacterial endotoxins.

Liver macrophages have strong plasticity; hence it can display different phenotypes with different stimuli.^[10]^ Due to those characteristics of hepatic macrophages (huge quantity, special location, plasticity), it participates in both maintain liver hemostasis and the development of various liver diseases. In steady state, KCs can induce immunological tolerance. As a kind of antigen presenting cells, KCs shows low levels of histocompatibility complex (MHC)-II, comparing CD11c + CX3CR1 + liver dendritic cells. What’s more, KCs possesses higher expression of the immune-suppressive costimulatory marker, programmed death ligand 1(PDL-1) than kidney and spleen macrophages. Interestingly, KCs are able to promote the activation of regulatory CD4 + T cells to induce immune tolerance. Those immune tolerance induced by KCs can relief immune-related extrahepatic tissue injury (kidney). In the inflamed liver microenvironment, hepatic macrophages composition will be out of proportion (the number of monocyte-derived macrophages increases), which will impair KCs’ tolerogenic phenotype.^[11]^

Hepatic macrophages also participate in the progress of many liver diseases. KCs usually secrets chemokine (including IL-β, TNF-α) and chemokine (including CCL2, and CCL5), the latter of which can recruit monocytes, so that the number of hepatic macrophages increases rapidly. Infiltrated monocytes can transform their phenotype from Ly-6Chi to Ly-6C+, which is associate with pro-inflammatory and pro-fibrotic functions.^[2]^ It is KCs that play dual roles in the Ischemia/reperfusion-induced liver injury. Not only can KCs cause hepatocyte death, endothelial damage and leukocytes recruitment, but it also has a protective function. In the process of hepatic sterile inflammation, activated KCs can secret inflammatory cytokines and chemokines to recruit other inflammatory cells including monocytes to amplify the inflammatory signaling.^[12]^

In a word, hepatic macrophages are a group of cells with a wide heterogeneity including its origin, phenotype and function. It plays a critical role in the occurrence and development of liver diseases. So, it is worthwhile to concentrate on figuring out the role of hepatic macrophages in many diseases and target it for disease treatment.

## 3. Autophagy

The word “autophagy” comes from Greek meaning “eating of self,” created by Christian de Duve. Cells are in a state of constant self-renewal, so they need to digest old substances to provide raw materials for the production of new components. Two major degradation systems exist in eukaryotic cells including the lysosome and the proteasome. Autophagy is used to describe the process by which cytoplasmic material is transported to lysosomes and digested.^[13]^ There are 3 types of autophagy: macroautophagy, microautophagy, and chaperone-mediated autophagy. Macroautophagy requires the formation of autophagosomes to sequester substances in the cytoplasm, which then fuse with lysosomes and degrade the substances in them. Microautophagy engulfs and digests tiny cytoplasmic components through lysosomal membrane invagination. In the chaperone-mediated autophagy, proteins directly cross lysosomal membranes mediated by chaperones.^[13]^ In fact, what we often call autophagy refers to macroautophagy. Autophagy regulates adaptive metabolic responses and can be activated to supply amino acids during starvation.^[14]^ Further research found that these amino acids produced by autophagy were mainly used to synthesize proteins to meet the needs of starvation.^[15,16]^ Autophagy also plays an important role in processes of differentiation and development of mammalian, and in immunity including direct elimination of microbes, control of inflammation, antigen presentation, lymphocyte homeostasis and secretion of immune.^[17,18]^ In the process of controlling inflammation, autophagy targets on NOD-like receptor family pyrin domain-containing 3 protein (NLRP3) inflammasome activators, inflammasome components, IL-1β signaling to orchestrate the activation of NLRP3 inflammasome.^[19]^

Comparing with brain and other organs, liver has higher baseline autophagic flux. Autophagy plays an important role in maintain liver hemostasis and is involved in many liver diseases such as non-alcoholic fatty liver disease (NAFLD), fibrosis, hepatocellular carcinoma, hyperammonemia, viral infections.^[20,21]^ Hepatocytes cells with autophagy defects are not able to eliminate cellular byproducts and prone to malignant transformation.^[22,23]^

## 4. Polarization and inflammasomes are 2 important processes regulated by autophagy

Polarization and inflammasomes can be visualized as a process by which cells are activated by internal and external stimuli, respectively. Hepatic macrophages realize their functions mainly through the release of various cytokine that is connect to 2 processes: polarization and activation of inflammasomes. Those 2 processes are closely relevant to liver diseases. Significantly, the autophagy is the upstream regulator of polarization and activation of inflammasomes.

### 4.1. Macrophages polarization

Polarization is used to describe the change of macrophages’ state due to the stimulus of pathogenic microorganism, cytokines, or some physicochemical factors. Their phenotypes are related to the microenvironment where macrophages locate.^[24]^ One of the purposes of macrophages biology is to make a thorough inquiry of polarization and its roles in physiological and pathological processes. In fact, the word “polarization” is similar to “activation.” Naive macrophages (Mφ or M0) can be activated to differentiate into 2 phenotype: M1 (classically activated macrophages) and M2 (alternatively activated macrophages).^[25]^ M1 is related to pro-inflammatory, which is activated by Th1 cytokines (IFN-γ and TNF-α), GM-CSF or lipopolysaccharide (LPS). Since then, those macrophages can secret pro-inflammatory cytokines including TNF-α, IL-1α, IL-1β, IL-6, IL-12, IL-23, COX-2, CXCL1~3, CXCL8-10, CCL2-5, CCL11, and low levels of IL-10.^[24,26]^ Though M1 possesses the ability of anti-microbial and anti-tumoral, it also promotes tissue damage and wound healing. M2 is characterized as anti-inflammatory, which produces anti-inflammatory cytokine such as IL-10, TGF-β, and IL-12, when stimulated by IL-4, IL-13, IL-33. M2 is able to clear debris and apoptotic cells, promote tissue repair and wound healing, and have pro-angiogenic and pro-fibrotic properties.^[26]^ M2 can be divided into other subtypes: M2a, M2b, M2c, and M2d.^[24]^

In summary, macrophages polarization means active state of macrophages in various organs. Polarization is the embodiment of macrophages’ plasticity. Macrophages mediate the initial and development of diseases by changing their active state. Once the state of M1/M2 polarization is out of control, homeostasis will be broken, leading to disease progression.

### 4.2. Inflammasome activation

Inflammasomes is a kind of multimeric protein complex that is assembled in the cell and mediates host protection against infection and physiological abnormalities.^[27]^ As we all know, the operation of the innate immune system relies on the recognition of microbial motifs by pattern-recognition receptors (PRRs) on the surface of immune cells such as macrophages, monocytes, dendritic cells and neutrophils. After PRRs distinguish various pathogen-associated molecular patterns, cytokine/chemokine and interferon can be produced to triggers pro-inflammatory and antiviral responses. Similarly, inflammasomes is multiprotein complex assembled after intracellular PRRs recognize pathogen-associated molecular patterns and danger-associated molecular patterns in the cytoplasm. This process promotes the maturation and secretion of interleukins (IL-1b and IL-18) that promote the inflammatory response to infection and injury.^[28]^

The canonical inflammasomes is compose of a cytosolic sensor (including NLRs, ALRs), an adaptor protein ASC and an effector pro-caspase-1.^[29]^ Once sensor detects specific stimuli, it can recruit ASC to form a multimeric complex. Afterwards, pro-caspase-1 can be recruited and changes into biologically active caspase-1 by self-cleavage. On one hand, caspase-1 subunits p20 and p10 cleaves pro-IL-1β and pro-IL-18 to IL-1β and IL-18. On the other hand, caspase-1 actives pore-forming gasdermin D to induce proptosis, whose character is cell swelling, lysis and release of contents.^[30]^ As a member of NLR family, NLRP3 is a classical inflammasome, activated in hepatic macrophages, which plays an significant role in sundry liver disease. In NASH, mitochondrial DNA (mtDNA) released from mitochondria can active NLRP3 in KCs.^[31]^ He et al^[32]^ found that suppressing NLRP3 in KCs can alleviate inflammatory and development of NASH. RIP1 kinase enhances the progress of NASH, by activating NLRP3 inflammasome in liver macrophages (hematopoietic-derived macrophages).^[33]^ HBeAg can inhibit the activation of NLPR3 in KCs to promote the HBV persistence and immune tolerance.^[34]^ Activation of NLRP3 in KCs contributes to liver fibrosis in schistosomiasis infection through via NF-κB.^[35]^

To sum up, activated inflammasomes especially NLRP3 in hepatic macrophages plays an important part in pathological process of liver diseases.

## 5. Autophagy of hepatic macrophages in liver diseases

**Figure 1.**
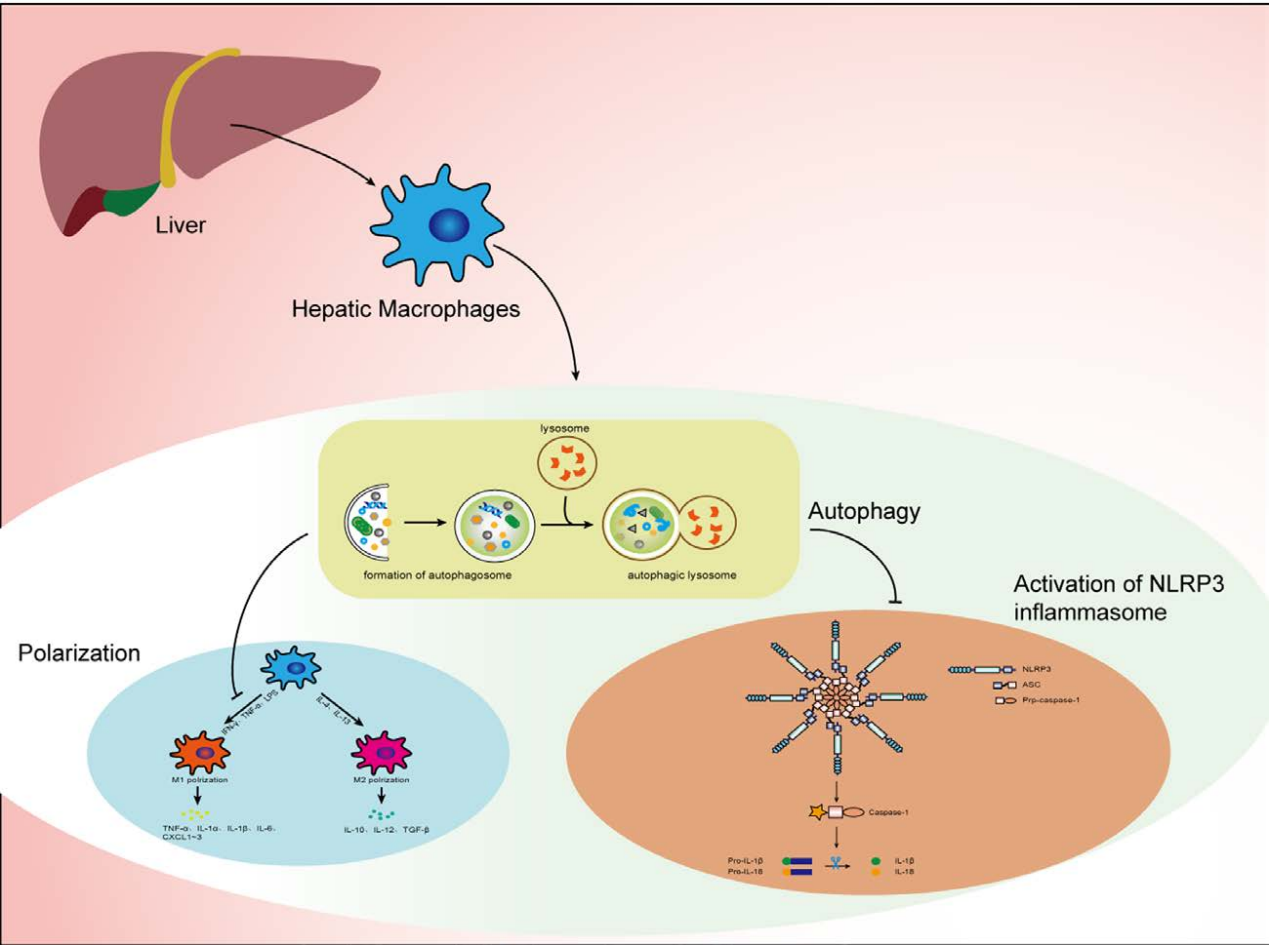
Many liver diseases are closely related to polarization of hepatic macrophage and activation of NLRP3 inflammasome in hepatic macrophage. The autophagy in hepatic macrophage can inhibit M1 polarization and activation of NLRP3 inflammasome, which can regulate the development of liver diseases and can be a target for the treatment liver diseases. NLRP3 = NOD-like receptor family pyrin domain-containing 3 protein.

Many liver diseases accompanied polarization and activation of inflammasomes in liver macrophages. For example, in NAFLD the excessively activated inflammation is caused by M1 polarized macrophages.^[36]^ In acute liver injury, KCs exhibit higher M1 polarization levels.^[37]^ On the other side, the activation of NLRP3 inflammasomes is common in NFALD.^[38]^ NLRP3 inflammasomes is also plays an important role in ischemia-reperfusion injury.^[39]^ In a word, the inflammation state of the liver is inseparable from polarization and NLRP3 inflammasomes of liver macrophages. The autophagy of hepatic macrophages can inhibit M1 polarization and activation of NLRP3 inflammasomes to regulate the liver diseases including NAFLD, liver injury, ischemia-reperfusion injury, hepatocellular carcinoma. (Table 1, Fig. 1).

**Table 1:** The autophagy regulating polarization and inflammasomes in liver diseases.

Diseases	Cell types	Regulation	References
Regulatory polarization			
HFD-induced obesity	Monocyte-derived macrophages, Kupffer	Autophagy deficiency→M1(p)M2(i)	^[40]^
ALI (TAA)	Kupffer	Enhancing autophagy→M1(i)M2(p)	^[41]^
Liver injury (APAP)	Kupffer	Enhancing autophagy→M1(i)M2(p)	^[42]^
ALI (TAA)	Kupffer	Inhibiting autophagy→M1(p)M2(i)	^[43]^
ALI (TAA)	Monocyte-derived macrophages	Autophagy deficiency→M1(p)	^[44]^
HCC	Monocyte-derived macrophages	Enhancing autophagy→M2(p)	^[45]^
HCC	Monocyte-derived macrophages	Enhancing autophagy→M1(p)	^[46]^
Regulatory inflammasome			
NAFLD (HFD)	Kupffer	Restoring autophagy flux→NLRP3(i)	^[47]^
NAFLD	Monocyte-derived macrophages	Autophagy activation→NLRP3(i)	^[48]^
ALD	Monocyte-derived macrophages	Autophagy deficiency→IL-1β, inflammasome(p)	^[38]^
ALI (CCL4)	Kupffer	Inhibiting autophagy→NLRP3(p)	^[49]^
ALI (GalN/LPS)	Monocyte-derived macrophages, Kupffer	Autophagy deficiency→IL-1β, inflammasome(p)	^[50]^
ALI (CCL4, LPS)	Kupffer	Enhancing autophagy→NLRP3(i)	^[51]^
IRI	Kupffer	Enhancing mitophagy→NLRP3(i)	^[52]^
IRI	Kupffer	Enhancing autophagy→NLRP3(i)	^[39,53,54]^

ALD = alcoholic liver disease, ALI = acute liver injury, APAP = acetaminophen, CCL4 = Carbon tetrachloride, GalN = D-galactosamine, HCC = hepatocellular carcinoma, HFD = high-fat diet, i = inhibit, IRI = ischemia-reperfusion injury, LPS = lipopolysaccharide, NAFLD = nonalcoholic fatty liver disease, P = promote, TAA = thioacetamide.

### 5.1. NAFLD

NAFLD is a kind of chronic liver disease related to obesity, insulin resistance, type 2 diabetes mellitus, hypertension, hyperlipidemia, and metabolic syndrome. Mild NAFLD is just characterized by simple steatosis, while sever NAFLD is also called NASH that will progress to cirrhosis or hepatocellular carcinoma. Depletion of KCs can reduce the level of serum TNF-α and relief liver injury in methionine-choline-deficient diet mice model.^[55]^ To make out the role of autophagy in activated KCs, KCs from Atg7-KO mice were stimulated with LPS, which showed that it released about 1.5 times TNF-α of cells from wild-type mice. Autophagy deficiency in KCs can enhance its sensitivity to LPS (increased TNF-α) by enhancing TLR signaling molecules, which contribute to the development of NASH.^[56]^ Atg5 knockout mice, treated with HFD to induce obesity model, suffers from systemic and hepatic inflammation under the stimuli of LPS. It is damaged autophagic flux in bone marrow-derived macrophages and KCs that increase proinflammatory M1 and decrease anti-inflammatory M2 polarization in both protein and gene aspects. Hence, autophagy deficiency in hepatic macrophages may lead their abnormal polarization (enhancing M1, inhibiting M2), which will cause hepatic inflammation and the progression to liver injury.^[40]^ Using free fatty acid and LPS can significantly down-regulate the expression of Foxo3a, which can lead to the activation of NLRP3 inflammasome through dampening autophagy. Liu et al proposes that improving Foxo3a-Bim transcription-facilitating autophagy flux-inhibiting NLRP3 inflammasome activation axis can be a target for treating NAFLD.^[47]^ The feature of NASH is reduced autophagy flux and activated inflammasomes. Ezetimibe, a blocking agent of cholesterol transporter NPC1L1, can attenuate steatohepatitis by promoting autophagy through AMPK activation and TFEB nuclear translocation. Furthermore, ezetimibe could inhibit NLRP3 inflammasome-IL1β pathway depending on autophagy and extracellular vesicles from hepatocytes. The hypercholesterolemia-treating drug Ezetimibe may have the potential to treat steatohepatitis and fibrosis.^[38]^ Innate immune response in NAFLD is closely related to polarization and polarization of hepatic macrophages, which can be regulated by autophagy. Enhancing autophagy will be an effective measurement to ameliorate inflammation state in NAFLD.

### 5.2. ALD

In 2014, the proportion of men and women who died from alcohol was 7.6% and 4.0% respectively.^[57]^ ALD is a kind of liver disease caused by chronic excessive alcohol consumption, whose spectrum ranges from simple fatty liver to more server cirrhosis and hepatocellular carcinoma (HCC). KCs has been found to contribute to the development of ALD. The activation of cannabinoid CB2 receptors (CB2) in KCs can inhibit M1 polarization through improving the expression of heme oxygenase-1 (HO-1). Then the KCs with the character of M1 polarization inhibiting can prevent the lipid accumulation in hepatocytes.^[58]^ CB2mye-/- mice show severer inflammation and steatosis caused by alcohol. CB2 agonist obstructed alcohol-induced liver inflammation and steatosis in wild-type mice, but it loses its effects in ATG5mye-/- mice. So, there is a link between CB2 and autophagy in anti-inflammatory. It has been proved that activation of CB2 receptor can relief steatosis and inflammation caused by alcohol, depending on activating autophagy in KCs.^[48]^

Autophagy can remove cellular waste to maintain hemostasis, while in ALD miR-155 can destroy autophagic flux by lowering the expression of lysosomal-associated membrane protein 1 (LAMP1) and LAMP2. Interestingly, the impairment of autophagy can induce the production of exosomes. The level of miR-155 in exosomes, secreted from KCs and hepatocytes treated with alcohol, increases, which may be a method of cell self-rescue.^[59]^ So atypical exosomes secretion can compensate for lysosomal dysfunction to maintain homeostasis in ALD. Mice with macrophages autophagy deficiency are prone to getting hepatic and systemic inflammation initiated by innate immune response, when administered with ethanol and LPS. The underlying mechanism is that autophagy deficiency in macrophages will improve the production of IL-1β arising from inflammasomes activation, rather than increasing the number of hepatic inflammatory cells.^[60]^ Therefore, autophagy in hepatic macrophages paly a protective role in ALD, it can remove cellular waste. What’s more, exosomes may counter autophagy breakage to clear up harmful substances. Macrophages’ autophagy can relief liver injury caused by alcohol partly depending on repressing Inflammasome activation.^[60]^ Increasing autophagy may be a promising therapy for ALD. It is puzzling that activation of autophagy through CB2 receptor can relief steatosis, while Ghulam Ilyas et al pointed out the autophagy decreasing do not affect the hepatic steatosis.^[48,60]^ To what extent the autophagy in hepatic macrophages influence hepatic steatosis and what cause the difference in conclusions deserve further study.

### 5.3. Acute liver injury (ALI)

As we all know, the blood of the digestive tract finally flows into the liver through the portal vein. The liver is the biochemical factory of the human body and plays a role in catabolizing the substances absorbed by the gastrointestinal tract. Due to its physiological function and anatomy, the liver is susceptible to damage by harmful substances.^[61]^ At the same time, because of its location in the liver sinusoids and its role in maintaining homeostasis, KCs are known as the gatekeepers of the liver. Numerous articles related to the functions of KCs in acute liver injury have published.^[62]^ Polyamines, as a regulator of cell growth, cell differentiation, and synthesis of DNA, RNA, and proteins, can alleviate carbon tetrachloride, ethanol and ischemia/reperfusion inducing liver injury. Zhou et al found that spermine (one of polyamines) can promote autophagic flux by improving the expression of ATG5, which could curb the M1 polarization and facilitate M2 polarization of KCs to attenuate ALI associated to TAA.^[37]^

In addition to multi-differentiation potential, mesenchymal stem cells also have immunomodulatory functions.^[44]^ In order to extenuate the ALI caused by Acetaminophen, human amniotic mesenchymal stromal cells can inhibit M1 polarization and enhance M2 polarization by promoting autophagy in KCs.^[42]^ Senescence can lead to the suppression of autophagy through inhibiting ATG5. Aged macrophages are prone to M1 polarization, compared to young macrophages. In TAA induced ALI, the restoration of autophagy in aged BMDMs will change its polarization state (resemble M2 polarization) that protect liver from injury. Concentrating on autophagy in macrophages may be a tactic to counter dysfunction caused by senescence.^[41]^

In acute toxin-induced liver injury (GalN/LPS induced), autophagy inhibition can lead to the release of IL-1β in both KCs and monocyte-derived macrophages. Importantly the increase of IL-1β is not relevant to its mRNA levels, but corresponding to inflammasome activation which facilitates cleavage of pro-IL-1β.^[50]^ KCs in TAA-treated ALI mice with diabetes mellitus display the trait of NLRP3 inflammasome activation that leads to severer liver injury than TAA-treated ALI mice without diabetes mellitus. The reason is that hyperglycemia can inhibit autophagy through AMPK/mTOR signaling pathway, which further enhancing the formation of NLRP3 inflammasome.^[43]^

Quiescent hepatic stellate cells (HSCs) are able to store retinoids (vitamin A) in its droplets. However, when HSCs is active, it will lose the ability to store droplets and participate in fibrosis. All trans retinoic acid (ATRA), as main metabolite of vitamin A, is also one of important substance stored in HSCs.^[63]^ Interactions between KCs and HSCs play an important role in ALI. In ALI model caused by CCl4, HSCs can synthesis and secret more ATRA, accompanied by the activation of HSCs. Next, ATRA can inhibit the autophagic flux through AKT/mTOR pathway in KCs, which lead to the accumulation of ROS. ROS will result in the activation of NLRP3 inflammasome, which induces the release of IL-1β and pyroptosis. Hence, the liver injury in ALI can partially attribute to that HSCs affect the autophagy in KCs.^[49]^

AMPK/mTOR signaling pathway may play a central role in restrain autophagic flux in KCs. TAM (TYRO3, AXL, and MERTK) family of receptor tyrosine kinases, expressing in macrophages, has a function of curbing inflammation. In LPS and CCl4 induce ALI, GAS6-AXL signaling can enhanced autophagy in KCs, which will curb the formation of NLRP3 inflammasome to limit the release of proinflammatory cytokines including IL-1β and IL-18. It is worth noting that the signal transmission is dependent on autophosphorylation of 2 tyrosine residues and the existence of MAPK14.^[51]^ T-cell immunoglobulin and mucin domain-containing molecule-3 (Tim-3) existing in the surface of monocytes and other immune cells, will drop from monocytes under the LPS, which can from sTim-3. In liver failure, sTim-3 plays a protective role. First, sTim-3 can inhibit the secretion of inflammatory mediators such as HMGB1, by promote the autophagy in monocytes. sTim-3 can also decrease the number of Ly-6C + monocytes (related to pro-inflammatory) and inhibit the infiltration of monocyte-derived macrophages in the liver of D-GalN/LPS model.^[64]^ In ALI, autophagy paly a protective role. On one hand, autophagy can inhibit M1 polarization and promote M2 polarization of liver macrophages. On the other hand, autophagy can downregulate the activation of NLRP3 inflammasome. In order to manipulate the autophagy of hepatic macrophages, AMPK/mTOR signaling pathway can be a target.

### 5.4. Hepatic ischemia‑reperfusion injury (HIRI)

Ischemia-reperfusion injury refers to the aggravation of damage to organs after the restoration of blood and oxygen supply. HIRI of liver is usually connected to partial hepatectomy, liver transplantation, hypovolemic shock and trauma. KCs mediate the liver injury in HIRI by producing reactive oxygen species and proinflammatory mediators.^[65]^ Suberoylanilide hydroxamic acid (SAHA) can alleviate orthotopic liver transplantation-induced HIRI in 2 ways, which depends on the existence of KCs. First, it can restrict the M1 polarization of KCs through AKT/GSK3β/NF-κB pathway. On the other hand, SAHA is able to induce autophagy via inhibiting AKT/mTOR signaling.^[66]^

Mitophagy, a type of microautophagy, is usually caused by mitochondrial depolarization, proteotoxic stress, dysregulated calcium signaling and molecular oxygen deficiency, which is an important way that cells selectively eliminate their own mitochondria to maintain their homeostasis. PTEN-induced kinase 1 (PINK1)/Parkin is regard as the main regulator of ubiquitin-dependent mitophagy. In HIRI, reinforcing the expression of PINK1 can promote the autophagy of KCs, which further inhibits the activation of NLRP3 activation to relief the liver injury caused by KCs.^[52]^ Eva-1 homologous gene A (Eva1a) is a kind of lysosomal and endoplasmic reticulum-related protein related to autophagy and apoptosis, whose expression can increase in KCs during HIRI. Knockdown Eva1a in KCs can inhibit the formation of autophagosomes, which will aggravate inflammation in liver Ischemia-reperfusion through activating NLRP3 inflammasome. More specifically, Eva1a can orchestrate autophagy by prolonging autophagosome membrane depending on stimulating ATG12/ATG5 complex.^[39]^ Because the expression of Eva1a improves in liver Ischemia-reperfusion, it may be a self-protection method to help liver immunity from HIRI. Lycopene can enhance autophagy via activating Nrf2/HO-1 pathway and further restrict the activation of NLRP3 inflammasome in KCs to moderate the HIRI. It is worth noting that autophagy downregulates the mRNA and protein levels of NLRP3, ASC and IL-1β to dominate the formation of inflammasome.^[53]^ Vacuole ATPase (V-ATPases) is electronic proton pump on eukaryotic cell organelles and plays an important role in the formation of acidic pH, which is indispensable to lysosomal degradation. The expression of ATP6V0D2 (V-ATPase D2 subunit) in macrophages increases in HIRI and the improvement of ATP6V0D2 can enhance the autophagic flux to curb the formation of NLRP3 inflammasome.^[54]^

Obviously, autophagy activation plays a beneficial role in liver ischemia-reperfusion injury. The induction of autophagy can be exogenous such as SAHA and lycopene, and it can also be the self-substance of liver macrophages to promote autophagy such as Eva1a and ATP6V0D2. In brief, autophagy of liver macrophages regulates HIRI through 2 ways: inhibiting M1 polarization and downregulating the activation of NLRP3 inflammasome.

### 5.5. Pathogenic microorganism infection

After infection of schistosomiasis japonicum and schistosomiasis mansoni, parasite eggs can cause granulomatous inflammation of the liver that eventually leads to liver fibrosis, portal hypertension, hemorrhage, and even death. Macrophages account for 30% of granuloma cells, which are recruited to wrap schistosome eggs. In S. japonicum-infected mice, autophagy inhibition caused by IL-7 via AMPK pathway regulates the host immune responses including increasing the number of liver macrophages and promoting the granuloma development and collagen deposits.^[67]^ During mycobacterium tuberculosis (Mtb) infection, alveolar macrophages cannot completely eliminate Mtb and become the niches for Mtb. On the contrary, KCs can effectively control the growth of Mtb. By comparing the metabolites of KCs and alveolar macrophages infected with Mtb, it was found that KCs express higher levels of ornithine and imidazole, which affect the growth of Mtb. Ornithine mediate autophagy via AMPK to restricts the growth of Mtb. Meanwhile imidazole wipes out Mtb by reducing the activity of cytochrome P450. This study clarifies the mechanism of KCs against Mtb and provides new ideas for the treatment and defense of Mtb.^[68]^

In an LPS-induced sepsis, superparamagnetic iron oxide nanoparticles can promote autophagy through Cav1-Notch1/HES1 signaling pathway to enhance the secretion of IL-10 in macrophages, and further alleviate inflammation and injury in liver.^[69]^

During microorganism infection, the autophagy of liver macrophages can enhance its ability to clear microorganism and promote the secretion of IL-10 to alleviate inflammation. Autophagy of liver macropahges not only helps to clear infection but also helps to control inflammation.

### 5.6. Hepatocellular carcinoma

Tumor-associated macrophages (TAMs) are macrophages involved in the formation of the tumor microenvironment and are derived from circulating monocytes. There is also evidence that tissue-specific resident macrophages are also a source of TAM.^[70]^ Like other macrophages, TAMs can be divided into 2 phenotypes, M1 and M2. M1 is characterized by tumor suppression, and M2 is closely associated with tumor cell proliferation, invasion, metastasis, and angiogenesis. Therefore, targeting TAMs can be a promising therapy for cancer.^[71]^ High‑mobility group box 1(HMGB1) from HCC increases the production of ROS depending on NOX2, which is capable of trigging autophagy through TLR2. The enhanced autophagy contributes to the M2 polarization of TAMs, bringing about the increased proportion of M2 macrophages in the tumor microenvironment. As mentioned before, M2 TAM promotes tumor cell growth, so the crosstalk between M2 TAMs formed a vicious circle. Attenuating the HCC growth by blocking ROS and HMGB1 could be a therapeutic target.^[45]^ In diethylnitrosamine‑treated mice model, the ATG5Mye-/- group was more likely to develop liver cancer and had a higher tumor burden and exhibits impaired immune cell recruitment and activation. More importantly, HCC cells can promote expression of programmed cell death‑ligand 1 (PD‑L1) in macrophages through destroying autophagy. Conversely, regulating autophagy with drugs can reduce programmed cell death-ligand 1 expression and promote the transition of macrophages to the M1 antitumor phenotype.^[46]^ In autophagy-deficient KCs, mitochondrial ROS can promote the production of IL1α/β to mediate liver inflammation and fibrosis via NF-kB-associated pathways, which play a decisive role in hepatocarcinogenesis during the preneoplastic stage.^[72]^

Overall, autophagy of hepatic macrophages has a dual role in hepatocellular carcinoma. The enhancement of autophagy can promote the M2 polarization of macrophages to promote the growth of tumor cells, meanwhile, inhibition of autophagy can promote the expression of programmed cell death-ligand 1 and leads to immunosuppression. In addition, autophagy deficiency of KCs can lead to liver inflammation which is related to formation of HCC.

### 5.7. Other liver diseases

Sirtuin 1, a multifaceted histone deacetylase, is upregulated in the liver macrophages of bile duct ligation mice and overexpression of which can cause the inflammation in cholestasis. The mechanism is that Sirtuin 1 can enhance the activity of NLRP3 inflammasome and inhibit autophagy through mTORC1, which lead to the activation of macrophages. What’s more, transplantation of bone marrow cells overexpressing SIRT1 enhances liver injury and fibrosis in a mouse model of cholestatic disease.^[73]^ Although the study did not mention the regulation of autophagy on inflammation in cholestasis, differential expression of SIRT1 could affect both autophagy and inflammasomes activation. This co-altered signature suggests a possible link between autophagy and inflammasomes activation.

With the development of technology, nanomaterials have been gradually applied to biological imaging, drug delivery, tissue repair and regeneration, and cancer treatment.^[74]^ Interestingly, nanomaterials are prone to accumulate in liver, which can be as a carrier for targeted drug delivery and cause damage to liver. Upconversion nanoparticles (UCNs), a potential imaging nanomaterial, can depletes KCs to induce liver injury. In fact, KCs can play a protective role, because the inhibition of autophagy is beneficial to KCs’ survival that further prevent UCNs to disturb to hepatocytes to initiate nanotoxicity.^[75]^ In most situations, the autophagy of liver macrophages can regulate inflammation and thus play a protective role, but excessive autophagy in UCN-engulfing KCs affects cell survival and promotes nanoparticle release.

## 6. Conclusion

Inflammation play an important role in liver diseases such as NAFLD,^[36]^ ALD,^[60]^ ALI,^[37]^ HCC,^[72]^ which is closely connected to polarization of hepatic macrophages and activation of inflammasomes. Intriguingly, autophagy in hepatic macrophages can regulate polarization and activation of inflammasomes. Hence, autophagy in hepatic macrophages can play dual role: a regulator of liver disease and a therapeutic targe for liver diseases.

This regulatory process is complex and diverse. First, the regulators of hepatic macrophages’ autophagy come from a variety of sources. They can come from inside the cell, like Eva1a and ATP6V0D2.^[39,54]^ Other cells and drugs can also control autophagy of hepatic macrophages such as HSC,^[49]^ hepatic carcinoma cell,^[45]^ empagliflozin, ezetimibe,^[38]^ and lycopene.^[53]^ Secondly, the downstream regulation of autophagy is also diverse. For example, in the process of regulating inflammasomes, autophagy can downregulate the mRNA and protein levels of NLRP3,^[53]^ ASC and IL-1β, while it can just affect cleavage of pro-IL-1β.^[50]^ Therefore, it is a big challenge to clarify the regulatory process of autophagy.

In the liver diseases, autophagy plays a double-edged sword role. Autophagy in liver fibrosis is necessary for trans-differentiation of HSCs, which contribute to the fibrogenesis. Autophagy in macrophages can also limit liver fibrosis through antioxidant and anti-inflammatory.^[21]^ Interestingly, Autophagy inhibits the transition to malignancy before HCC occurrence.^[76]^ While in established HCC, autophagy conduces to tumor progression, metastasis and resistance to therapy.^[23]^ Similarly, autophagy of hepatic macrophages plays a dual role in inflammation-related diseases, such as NAFLD, ALD, HIRI, and the activation of autophagy in hepatic macrophages show effects of inhibiting inflammation and relieving disease development. On the other hand, in liver injury caused by UCNs, excessive autophagy of hepatic macrophages can aggravate liver damage, which could make more UCNs disturbed into hepatocytes.^[75]^ In HCC, the cancer cells can facilitate the M2 polarization of macrophages, which provide an immunosuppressive microenvironment for tumor cells.^[45]^

As a regulator, autophagy in hepatic macrophages can be a therapeutic target. Several studies have shown that the decrease of macrophage autophagy is an important step in the pathogenesis of disease. Many researchers have tried to control the autophagy in hepatic macrophages to relief liver injury in many liver diseases. Liu et al upregulated the autophagy flux in KCs by altering the expression of Foxo3a, which can alleviate the inflammation in NAFLD.^[47]^ Ezetimibe was also found to induce autophagy in hepatic macrophages to block the activation of NLRP3 inflammasome, which will furtherly relief steatohepatitis.^[38]^ Spermine and human amniotic mesenchymal stromal cells can facilitate M2 polarization and inhibit M1 polarization, which will ameliorate liver injury.^[37,42]^ In HIRI, many molecules were found to regulate hepatic macrophage’s autophagy, such as PINK1, Eva1a, ATP6V0D2.^[39,52,54]^ Altering the expression of those molecules can alleviate HIRI by regulating autophagy. In a word, the inflammation is the cause of liver damage in many diseases. Meanwhile the M1 polarization and activation of inflammasomes are 2 sources of inflammation. Autophagy in macrophages can function as a regulator of polarization and inflammasomes. However, the M2 polarization of TAM can be an accomplice to HCC. Inhibiting autophagy in TAM to block M2 polarization may be a measure to treat HCC. Although the number of autophagy studies in liver macrophages is limited, with the deepening of research, autophagy in hepatic macrophages is expected to become a target for disease treatment.

Although the disease occurs as a result of the joint action of multiple cells, macrophages play an important role in the process of liver diseases. In this review, we focus on the role of autophagy in hepatic macrophages. Understanding the mechanism and effect of autophagy in macrophages in the liver may bring new insight into effective management of the liver diseases.

## Acknowledgments

The authors want to thank The 306th Hospital of PLA-Peking University Teaching Hospital, Beijing, China for providing resources for completion of the current article. We acknowledge all authors whose publications were included in our article.

## Author contributions

**Conceptualization:** Yan Cui.

**Supervision:** Yuan Yue, Sheng-Yu Lu, Hong-Yun Nie, Tao Zhang, Pei-Ming Sun, Hong-Feng Yan, Hong-Wei Sun, Jian-Wu Yang, Jin-Lian Zhou.

**Validation:** Yan Cui, Hao Li, Sheng-Yu Lu.

**Visualization:** Yan Cui, Hao Li, Jia-Qi Yang, Yuan Yue, Sheng-Yu Lu, Hong-Yun Nie, Tao Zhang, Pei-Ming Sun, Hong-Feng Yan, Hong-Wei Sun, Jian-Wu Yang, Jin-Lian Zhou.

**Writing – original draft:** Jun Ge.

**Writing – review & editing: Jun Ge, Hao Li, Yan Cui**.
